# Recent progress of fiber-based transistors: materials, structures and applications

**DOI:** 10.1007/s12200-022-00002-x

**Published:** 2022-03-29

**Authors:** Haozhe Zhang, Zhe Wang, Zhixun Wang, Bing He, Mengxiao Chen, Miao Qi, Yanting Liu, Jiwu Xin, Lei Wei

**Affiliations:** School of Electrical and Electronic Engineering, Nanyang Technological University, Singapore, 639798 Singapore

**Keywords:** Electronic textile (e-textile), Fiber-based transistor, Logic computation, Sensing, Fiber-based memory device

## Abstract

Wearable electronics on fibers or fabrics assembled with electronic functions provide a platform for sensors, displays, circuitry, and computation. These new conceptual devices are human-friendly and programmable, which makes them indispensable for modern electronics. Their unique properties such as being adaptable in daily life, as well as being lightweight and flexible, have enabled many promising applications in robotics, healthcare, and the Internet of Things (IoT). Transistors, one of the fundamental blocks in electronic systems, allow for signal processing and computing. Therefore, study leading to integration of transistors with fabrics has become intensive. Here, several aspects of fiber-based transistors are addressed, including materials, system structures, and their functional devices such as sensory, logical circuitry, memory devices as well as neuromorphic computation. Recently reported advances in development and challenges to realizing fully integrated electronic textile (e-textile) systems are also discussed.

**Graphical Abstract:**

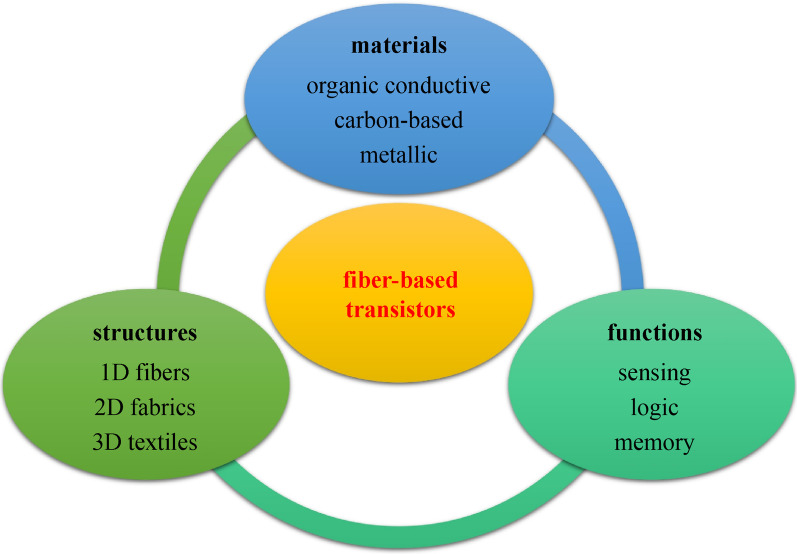

## Introduction

Electronic devices have gone through significant developments, whose functions are achieved by combining various materials with designed alignments and structures. These devices have dramatically changed our lives, through connecting people around the world and enabling the interaction between electronic devices and the environment. Wearable electronics, a new category of electronic device, can be constantly worn as accessories, embedded in clothes, or adhered to the human body to enhance the direct interaction between the user and the surroundings. Nowadays, some wearable devices, such as smartwatches, smart bands, smart glasses, and wearable AR/VR, are available in the market and favored by the public. On the other hand, clothes made from fabrics or textiles provide attractive properties, such as high dynamic bending elasticity and stretchability while retaining high strength and permeability, light weight potential, and thermal and chemical stability. The most promising property of such fabrics is the high possibility of engineering material composition at the right scales in proper architectures along lengths of a kilometer [1, 2]. Moreover, benefiting from the development of micro- and nano-manufacturing technology, the feature size has been reduced to several nanometers scale, which enables multi-functionality integration. The integration of sensing, power harvesting, actuating, calculation and display enable systematic programming and analysis leading to reaction to external stimuli [3]. Previous studies have demonstrated that transistors, memory devices, organic light-emitting diodes, and energy storage devices have already been constructed on curved surfaces and flexible substrates [4]. However, in most cases, these devices operate as individual units and they are incapable of strong interaction with each other, thus decreasing the potential to undertake complex tasks such as complicated computations, machine vision, and effective control systems.

**Fig. 1:**
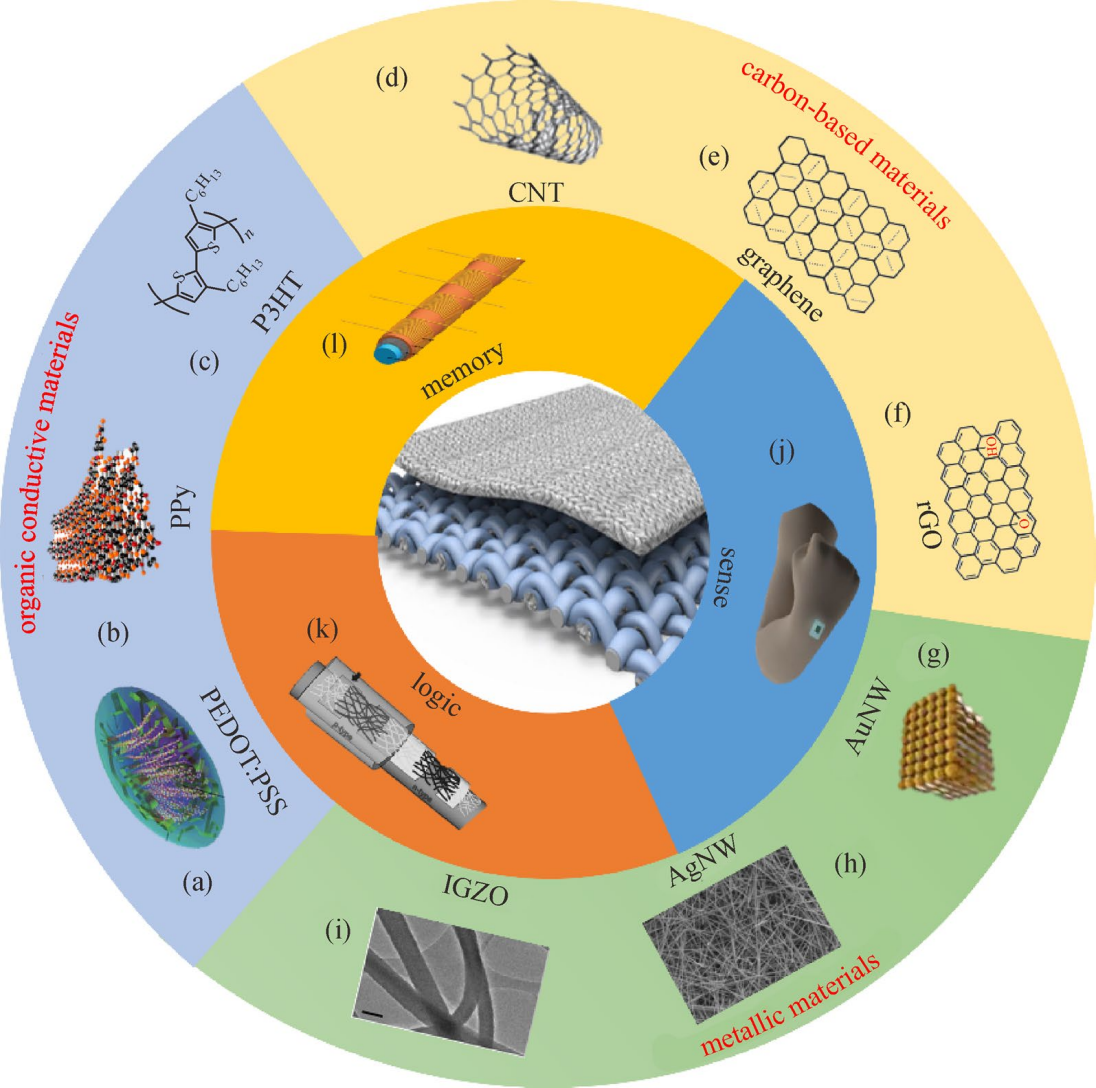
Summary of accessible materials and their applications in sensing, memory, and logic computing. **a** Colloidal gel particle of PEDOT:PSS. Reproduced with permission [7]. Copyright 2015, Springer Nature. **b** Schematic of PPy nanostructure. Reproduced with permission [8]. Copyright 2016, Elsevier. **c** Chemical structure of P3HT. Reproduced with permission [9]. Copyright 2016, Elsevier. **d**–**f** Carbon nanotube (CNT) nanostructure, graphene and rGO chemical structure. Reproduced with permission [10, 11]. Copyright 2018, MDPI. Reproduced under the terms of the Creative Commons Attributional license (CC BY 4.0). **g** Illustration of AuNW. Reproduced with permission [12]. Copyright 2020, Wiley–VCH. Reproduced under the terms of the Creative Commons Attribution license (CC BY 4.0). **h** Illustration of AgNW. Reproduced with permission [13]. Copyright 2012, Wiley–VCH. **i** TEM image of thermally processed IGZO. Reproduced with permission [14]. Copyright 2020, Springer Nature. Reproduced under the terms of the Creative Commons Attribution license (CC BY 4.0). **j** Fabricated strain sensor. Reproduced with permission [16]. Copyright 2020, Wiley–VCH. **k** Fiber-type CMOS circuitry. Reproduced with permission [16]. Copyright 2017, Wiley–VCH. **l** Illustration of fiber-shaped perovskite memristor. Reproduced with permission [17]. Copyright 2016, Wiley–VCH

To achieve a fully integrated electronic textile (e-textile) system, several approaches have been proposed, two of which have the highest level of universal recognition, namely: (1) embedding electronic devices directly in textiles or fabrics, (2) weaving the electronic fibers into textiles [5]. Although embedding electronic devices in fabrics is an easier method to realize electronic functions, the challenges of minimizing low-comfort wearability and improving poor compatibility between electronic devices and fabric substrate remain. Therefore, more research works are focusing on fiber-based functionalities, which are more suitable for large-area and complicated e-textile systems [1, 5]. High compatibility between fiber networks and the diversity of materials provides potential for a wide range of applications. From the previous studies, materials used to fabricate fiber-based devices can be roughly classified into three groups: organic materials, carbon-based materials, and metallic nanowires (NWs)/nanoparticles (NPs) [6], as summarized in Fig. 1. Organic conductive polymers, including poly (3,4-ethylene dioxythiophene) polystyrene sulfonate (PEDOT:PSS) [7, 18], poly (3-hexylthiophene)(P3HT) [9, 19], and polypyrrole (PPy) [8, 20], are broadly applied in fabricating wearable electronic components and devices due to their high flexibility, low density, ease of fabrication and widescale availability. They are normally classified as cation salts of the highly conjugated polymer [21], whose electrical properties can be fine-tuned by organic synthesis and by advanced dispersion techniques [22]. Their high processability allows large-scale manufacturing with low cost. However, their long-term stability remains to be improved. Carbon-based materials have been comprehensively investigated during the last decades and used in a large range of applications. Their chemical combinations with other organic materials and various elements form strong covalent bonds, thus exhibiting large surface area, high density, and high mechanical strength and hardness. Carbon nanotubes (CNTs) [10, 23], graphene [24], and reduced graphene oxide (rGO) [11, 25] have become important materials in aerospace, photovoltaics, electronics, energy storage, and harvesting. One major difficulty that limits the applications of carbon-based materials in mass production is the high price due to complex and costly preparation and fabrication [26]. Hybrid materials offer promising opportunity to overcome these limitations. In addition to organic materials and carbon-based materials, the low dimensional nanostructures of metals are attractive for fiber-based devices due to their ultrahigh electrical conductivity [12, 13]. Their ease of blending with organic conducting materials broadens the application possibilities. For instance, silver and gold plated fibers show improved performance as electrodes [18, 27]. Commonly, metallic materials exhibit high stiffness and brittleness, which limits their applications in wearable electronics. An alternative solution is to coat metallic materials on other commercial fiber surfaces by depositing and electrospinning to improve stretchability.

Based on these materials, many electronic devices integrated with textiles have been exhibited and have incorporated satisfactory adhesion to irregular surfaces and high tolerance to deformations caused by twisting or stretching, with acceptable electrical performance [5, 28]. Among all these devices, computation ability is the key parameter for smart textiles to process the incoming signals and execute complicated tasks. Modern integrated circuits composed of millions of transistors provide strong computational capacity in signal processing and data calculation. To further improve the integrity of chips, the study of transistors has been intense. In this paper, we mainly focus on fiber-based transistors in sensing, logic gates, memory, and neuromorphic computing as well as recent developments and challenges in e-textile systems.

## Fiber-based transistors

As one of the most important components in semiconductor devices, transistors are used to amplify or switch electronic signals in circuits, which allow a wide array of electronic devices. It is also the basis for circuit design, data transmission, and analysis. In e-textile systems, there are mainly two types of fiber-based transistors, namely, fiber field-effect transistors (FETs) and organic electrochemical transistors (OECTs).

### Fiber field-effect transistors (FETs)

A conventional planar thin-film transistor is typically fabricated by depositing an active semiconductor layer and a thin film of dielectric layer as well as metallic contacts on a supporting substrate and then micro-patterning through masking steps. However, the cylindrical shape of the fiber becomes a major challenge for uniform coating and precise patterning, which commonly degrades fabrication efficiency and device performance. In addition, some organic materials are not compatible with the necessary high-temperature processes, and this factor limits the formation of ultrafine microfiber during traditional micro-fabrication. To address these challenges, approaches that have been proposed include rotating the fiber during the metal deposition in vacuum and using organic materials with a solution-based approach, which provide an effective coating of multi-layers.

**Fig. 2:**
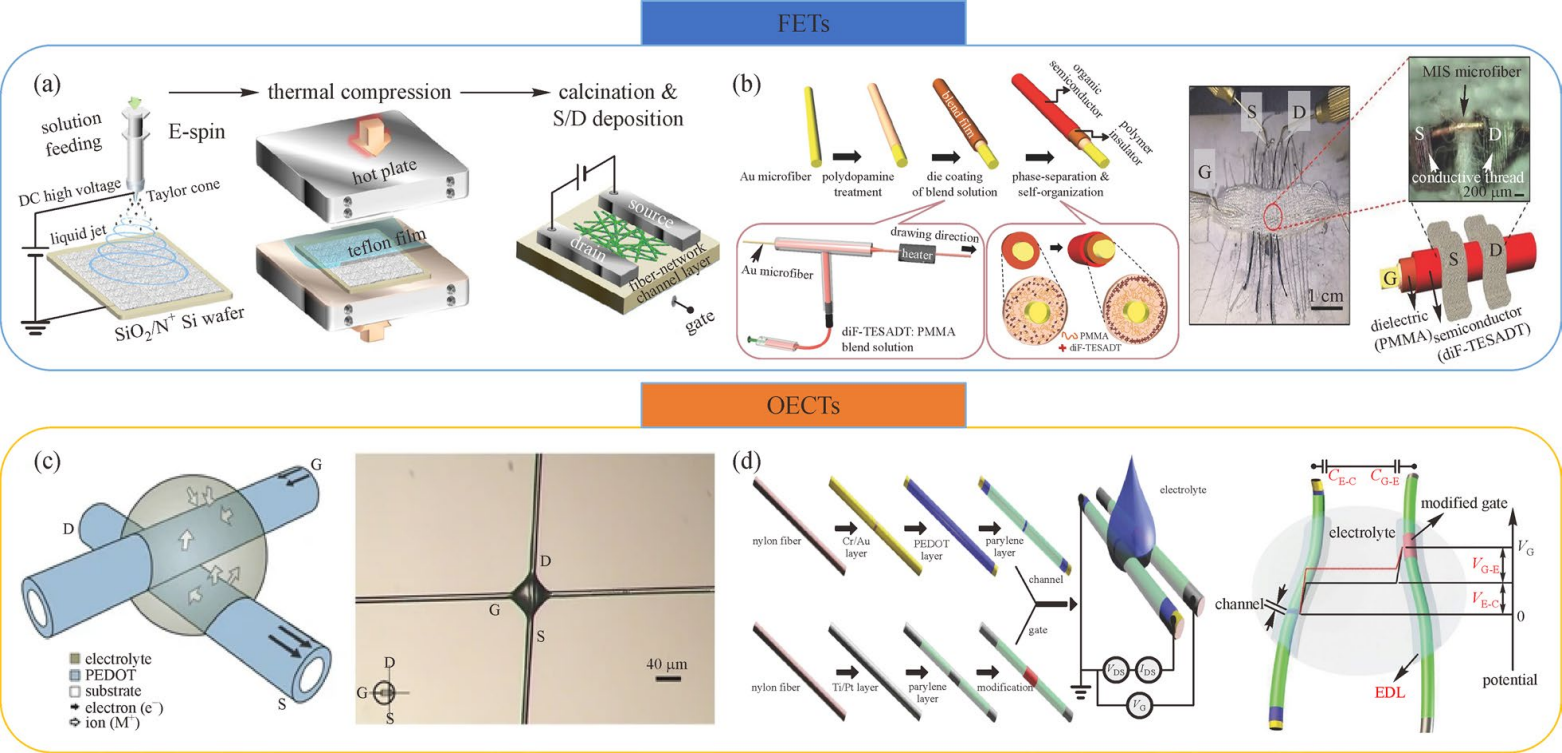
Fiber-based transistors. Schematic diagram of **a** fabrication steps and structure of composite fiber networks. Reproduced with permission [33]. Copyright 2014, American Chemical Society. **b** Fabrication process of MIS microfibers. Reproduced with permission [34]. Copyright 2016, Wiley–VCH. **c** A wire ECT. Reproduced with permission [35]. Copyright 2007, Springer Nature. **d** Functionalized fiber-based channel and gate for an OECT. Reproduced with permission [36]. Copyright 2018, Wiley–VCH

Benefiting from well-developed fabrication methods and high electrical performance of metallic materials, the deposition of the metallic layer can be a promising approach to fabrication of field-effect transistors on fibers. It has been reported that metallic oxides and NWs/NPs, such as indium gallium zinc oxide (IGZO) [14, 29–31], CuO [32], and Ag nanowires (AgNWs) [13] have been used on various substances. One solution has been to form a Cr/AlO_*x*_/IGZO/Al coaxial structure via a sputtering system, ALD system, dip coating, and thermal evaporation respectively. The resulting transistor offered relatively high mobility, large ON/OFF ratio, and a low leakage current with a high breakdown electrical field [30]. Another solution using IGZO as active channel material was reported by Park et al. [31]. Poly (vinyl alcohol) (PVA) was coated on bare fiber through dip-coating to reduce the surface roughness of the fiber, while Al_2_O_3_ and MgO nanolaminates enhanced insulating properties and offered superior mechanical properties. The field-effect transistor exhibited good mobility (over 3 cm^2^/(V · s)), low leakage current and off current of less than ${10}^{-9}$ and ${10}^{-13}$ A, respectively [31]. The incorporation between metal-oxide nanofibers (NFs) and field-effect transistors has great potential for nanowire or NF transistors [14, 33]. An example is the In_2_O_3_-ZnO-ZnGa_2_O_4_ composite NF network serving as channel layers as illustrated in Fig. 2a. Here, the composite fiber and the high-*k* dielectric layer were electro-spun and thermally deposited onto the SiO_2_/Si substrate [33]. The composite fiber mat provided superior electrical performance with field-effect mobility of ${\text{7 cm}}^{2} /({\text{V}} \cdot {\text{s}})$ and a low threshold voltage of about 1 V.

In addition to metallic materials and carbon-based materials, organic semiconductors (OSCs) have been used in a variety of applications [34, 37–39]. Kim et al. reported a metal-polymer insulator-organic semiconductor (MIS) coaxial microfiber as shown in Fig. 2b. Gold microfiber as gate electrode was coated by polydopamine (pDA) after surface modification which enabled the surface to be hydrophilic and significantly improved surface adhesion. Then, the pretreated fiber was passed through a T-shape glass tube filled with 2,8-difluoro-5,11-bis (triethylsilylethynyl) anthradithiophene (diF-TESADT) and poly (methylmethacrylate) (PMMA) blend solution and heated. Vertical phase separation of the polymer blend solution was utilized to separate PMMA and diF-TESADT, creating an Au/PMMA/diF-TESADT coaxial structure. With the pDA modification, the electrical performance showed a significant improvement, and the electrical field average mobility reached $(0.19 \pm 0.07)\; {\text{cm}}^{2} /({\text{V}} \cdot {\text{s}})$ with maximum mobility of ${\text{0.3 cm}}^{2} /({\text{V}} \cdot {\text{s}})$. The ON/OFF ratio was approximately ${{1}0}^{4}$ without hysteresis during voltage weep [34]. To address the high flexibility and stability of OSCs on the substrates, a deformable organic field-effect transistor has been proposed [40]. Uniform and continuous nanowires were formed by the OSCs composed of FT4 and DPP via electrospinning. Within this structure, the OSC NWs were placed on ${\text{SiO}}_{2}$/Si substrate with a high-*k* dielectric layer, demonstrating higher mobility of ${\text{8.45 cm}}^{2} /({\text{V}} \cdot {\text{s}})$ and deformability under large tensile and compressive strains than those on ${\text{SiO}}_{2}$ substrates [40].

Fiber-based FETs are one of the two most important components for developing advanced e-textile systems. Many materials and fabrication methods for building fiber FETs have been presented. These materials demonstrate admirable electrical properties. However, the challenges regarding environmental stability and mechanical performance remain to be solved.

### Organic electrochemical transistors (OECTs)

Due to the layer-by-layer structure of FETs, thermal deposition and mask patterning are always required, which complicates the fabrication process and prevents large-scale incorporation into textiles. Moreover, these transistors have a large operating voltage and are sensitive to the thickness of the gate dielectric layer, which further limits their electrical performance on fabrics. To overcome these drawbacks, different device structures were demonstrated on OECTs [35, 36, 41–46]. One typical structure was formed by placing two fibers coated with conductive materials in a cross geometry with a drop of solid electrolyte at the intersection. The overlap area and reversible process between the electrolyte and conductive fiber modulated the channel length and the conductivity of the effective channel respectively, which resulted in a change of several orders of magnitude, thereby realizing ON/OFF operation.

One OECT using PEDOT:PSS as the active layer was proposed by Hamedi et al., as shown in Fig. 2c, whose *I-V* characteristic was similar to that of P-type MOSFETs [35]. Ohmic or insulating contact could be accomplished by adding different materials into the junction at the crossing. Logic circuits such as inverter and multiplexer were presented, showing the possibility of realizing analog or digital circuits on the textile platforms. The same team also reported a P3HT and ionic electrolyte-based transistor, operating in both electrochemical and field-effect modes [42], enabling full electronic integration on woven textiles. However, electrolytes entered the channel due to capillary action, which might lead to uncertainty of channel length. To overcome this issue, a fixed-channel-length fiber-based transistor was developed. The vapor-coated nylon monofilament fiber was tightly stitched on a silk fabric pretreated with 1H, 1H, 2H, 2H-perfluorooctyltriethoxysilane, and the middle part of nylon fiber was fixed on the upper surface serving as the active channel while the other parts acted as the source and drain electrodes in the back surface, to determine the channel length [44].

High electrical conductivity can reduce the voltage drop along the entire length of the fiber, and this is critical for stabilizing the operation of electronic devices and decreasing power consumption. However, compared with metallic materials, organic materials always suffer from low electrical conductivity, which severely limits their electrical performance. To reduce the total impedance, Yang et al. [36] introduced metal/conductive polymer multilayer electrodes on fibers as shown in Fig. 2d, which presented high sensitivity, selectivity, and mechanical stability. Here, a Cr/Au/PEDOT:PSS multilayer was coated on nylon fiber, tremendously increasing the fiber conductivity. The resistance could withstand several thousand bending tests without significant increase. In the Cr/Au/PEDOT:PSS multilayer film, PEDOT:PSS also functioned as an adhesion layer to prevent cracking of the Cr/Au layer and consequent changes in fiber resistance. In another work, an all-in-one, fiber-shaped OECT was designed to reduce resistance and signal distortion [46]. Gold, PEDOT:PSS and parylene were coated on nylon fiber as electrodes, a channel layer, and insulator respectively. Meanwhile, CNT fiber was covered by Pt NPs and these fibers were twisted firmly. The *in-situ* amplification effect tremendously amplified the weak signal and made this OECT a biosensor with high sensitivity, dynamical stability, and anti-interference capability [46].

Benefiting from the interaction between the channel layer and the electrolyte, OECTs are insensitive to variations in local geometry or electrolyte patterns, thus providing highly flexible device geometry possibilities and reducing the operating voltage. Moreover, the simple fabrication processes and structures make OECT a strong candidate for large-scale e-textile systems. However, due to the electrochemical operation mechanism, OECT is only suitable for low-frequency operation at the current stage. Slow switching speed, low electrical performance, and long response time are still hindering the further development of OECT.

## Transistor-enabled functional textiles

### Wearable sensors

#### Biosensors

Biosensors are universally applied in multiple areas, such as clinical diagnosis, monitoring during treatment, and robotics. They are responsible for effectively selecting target analytes (for example, glucose, ions, and dopamine) and then transferring an electrical signal to the user interface, providing real-time health monitoring and biometric analysis [47]. With the rapid development of high-performance e-textile sensory systems, wearable biosensor technology is becoming a competitive candidate for medical sensing and assisting diagnostics due to their compatibility with human bodies, as well as their high accuracy, flexibility, and sensitivity. The micro and nanoscale fibers are very suitable for small-size tissue damage monitoring and testing [47]. As a consequence, use of fiber-based transistors as wearable biosensors has been widely adopted in recent decades, providing effective tools for healthcare monitoring.

Electrically conductive polymers are seen as promising materials for wearable biosensors for their high stretchability, simple fabrication, and excellent electrical properties. These materials include carbon nanotubes, graphene oxide, metallic nanoparticles, and nanowires [36, 48–53]. Wang et al. introduced one highly sensitive glucose monitoring system based on OECTs [49]. The precleaned raw PA6 filaments were wrapped through NF suspension and then polymerized to form the PPy/rGO/PA6 and PPy/rGO/PA6/GO_*x*_/nafion composites. After the NF was woven into a piece of textile, a drop of gel electrolyte was added into a cross-junction between two conductive fibers to complete the sensor architecture. The placement metallic gate electrode helped to achieve high sensitivity and quick response, which was critical for low concentration measurement. Due to the chemical reaction within the active layer, the channel current changed dramatically according to its concentration, thus enabling the analytes to be distinguished. Apart from glucose, dopamine measurement has been studied intensively [50, 51]. With a similar fabrication process, a PPy/NFs/PA6 structure was formed. At low gate voltage, the device showed an approximately linear current response with a sensitivity of 47.28 normailzed current response (NCR) per decade ranging from 1 nmol/L to 1 µ${\text{mol/L}}$ [50]. One Au-fiber based stretchable 3-electrodes sensor was proposed [53]. Previously, it was rather difficult to design biosensors with both high stretchability and sensitivity. Several pre-strained Au/SEBS fibers were plated with gold and divided into three groups: nonmodified Au fiber, Au/PB/GO_*x*_/Ch fiber, and Au/Ag/AgCl fiber as a counter electrode, working electrode, and reference electrode respectively. These helical fibers were weaved into a plain fabric in parallel, which enabled a large range of strain from 0 to 200% and achieved a linear range from 0 to 500 µ${\text{mol/L}}$ with glucose sensitivity of 11.7 µ${\text{A/((mmol/L)}} \cdot {\text{cm}}^{{2}})$ [53].

**Fig. 3:**
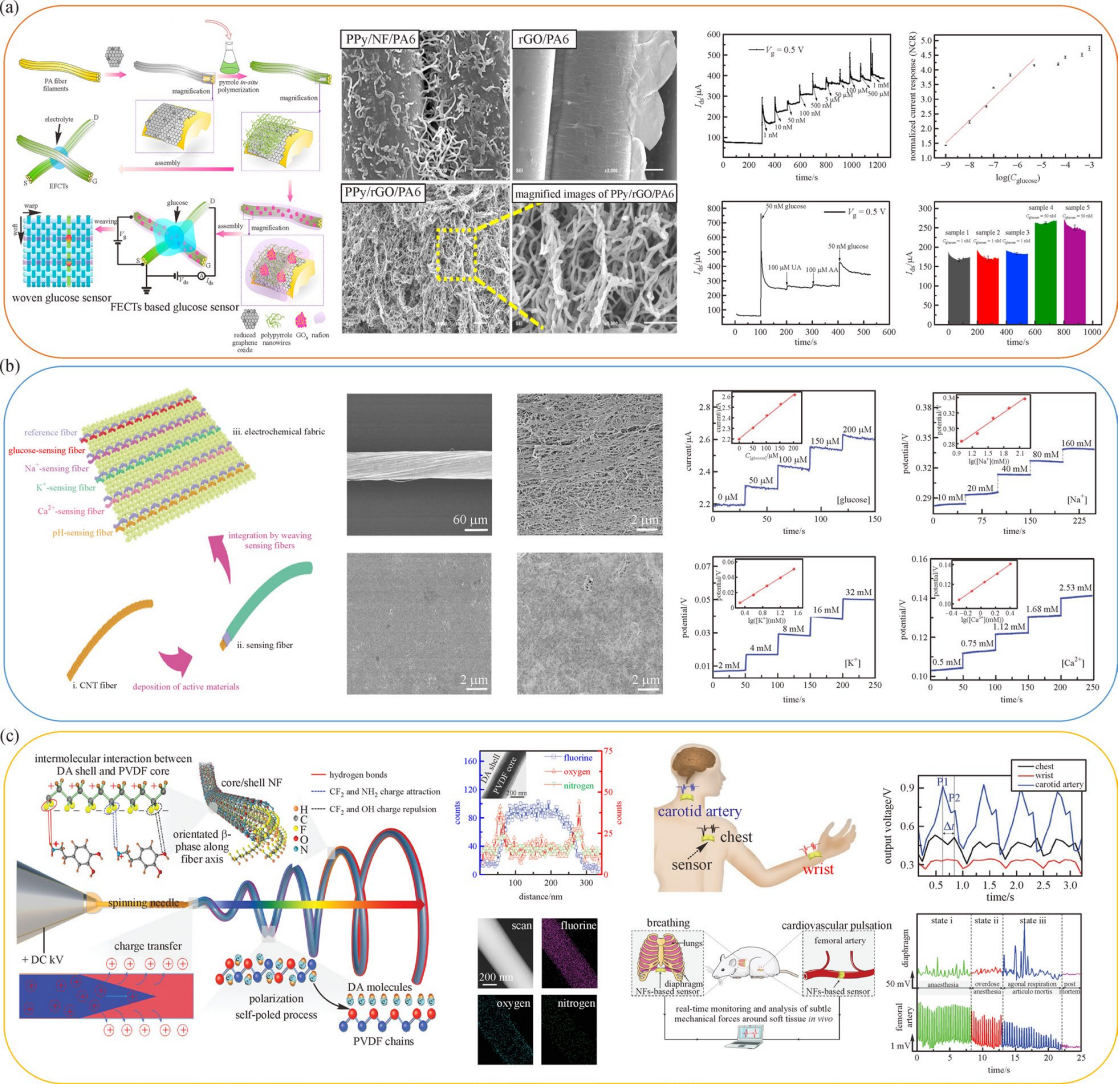
Schematic diagram of **a** glucose sensors fabrication and characterization, and glucose sensing ranging from 1 nmol/L to 1 mmol/L. Reproduced with permission [49]. Copyright 2017, Elsevier. **b** Fabrication of the electrochemical fabric by weaving sensing fibers and potential response to respective analyte solutions (glucose, ${\text{Na}}^{+}, \, {\text{K}}^{+}, \, {\text{Ca}}^{+},$ and pH). Reproduced with permission [55]. Copyright 2018, Wiley–VCH. **c** PVDF/dopamine nanofibers via electrospinning, voltage response according to blood pulsations and mice physiologic recording using the nanofibers. Reproduced with permission [58]. Copyright 2021, Wiley–VCH. 1 M = 1 mol/L

Being a common physiologic behavior of humans, sweating is considered as an important indicator of health state since it can convey physiologic information and is a suitable basis for non-invasive health monitoring [54]. Electrochemical fabrics have demonstrated highly effective and selective properties in sweat sensing in recent years [41, 52, 55–57]. Wang et al. demonstrated an integrated electrochemical sensor by weaving multiple sensing fibers into a single fabric, as shown in Fig. 3b [55]. All these sensing fibers were realized by depositing different materials onto CNT fiber, forming the fabric sensing block. The performance of these sensing fibers was characterized by different analyte solutions, showing the capability to monitor various analytes with high efficiency and replicability [55]. To conduct sweat analysis, the biosensors demand high selectivity and accuracy. By depositing ion-selective membranes on active material, a significant improvement in ion selectivity can be achieved, thereby providing more information about the specific kinetics of different ions [56, 57].

Apart from sweating, other physiologic behaviors also help the diagnosis of a healthy state. The measurement of physiologic pressures such as blood pressure and intraocular pressure is a common process in clinical diagnosis. A biomedical fiber sensor based on piezoelectric NF with a poly(vinylidene difluoride) (PVDF)/dopamine core/sheath structure via electrospinning was exhibited [58]. The interaction between PVDF and dopamine stabilized device performance and enhanced piezoelectric property. To further investigate the practical potential, the packaged sensor was tested on human bodies to measure blood pulsation, and then it was applied to mice to detect diaphragm motion and arterial pulsation as shown in Fig. 3c, proving the ability to identify diaphragm motions and blood pulsing at different physiologic states.

Although biosensing research and industry have made progress, there are still some challenges. The stability and robustness of the fabric sensors must be improved, while the concern of susceptibility to environmental influence should also be addressed. To promote the development of textile-based biosensors, device performance should be considered, but also the ability to analyze sensor data. Moreover, to accomplish predictive healthcare and to achieve high-level integration with a variety of functional blocks are being expected.

#### Strain/motion sensors

Besides biological and chemical sensing, various fiber-based sensors have been applied in strain and motion measurement. The conductive elements in these sensors are graphene [59–62], CNTs [63], rGO/thermoplastic polyurethane (TPU) [64], graphite [65], polymer/CNT composites [66, 67], and metallic nanowires [15, 68]. Besides, some reports indicated that with the aid of elastomer polymer substrates, such as polydimethylsiloxane (PDMS) [64, 66], Ecoflex [69], and polyurethane (PU) [70], the stretchability of the sensors was dramatically increased. Moreover, depending on the material properties, various fabrication methods have been proposed, such as layer-by-layer [59], dip-coating [70], wet-spinning [63, 71], and spray coating [72]. Several representative works are summarized in Table 1.

**Table 1:** Summary of strain/motion sensors on fabric: structures, materials, mechanisms, and properties

Configuration	Materials	Mechanism	Gauge Factor (GF)	References
Sheath-core	Graphite	Per-location	14.5 (15%)	[65]
2D	Carbonized silk	Cracks with strain	25 (0–80%), 64 (80–140%)	[69]
Sheath-core	MXene/PU	Piezoresistivity	12900 (152%)	[71]
2D	Graphene	Piezoresistivity	475 (14.5%)	[73]
Cross-geometry	Oxide/Ag NPs	Cracks with strain	15.2 (0–10%), 4.1 (10–150%)	[74]

**Fig. 4:**
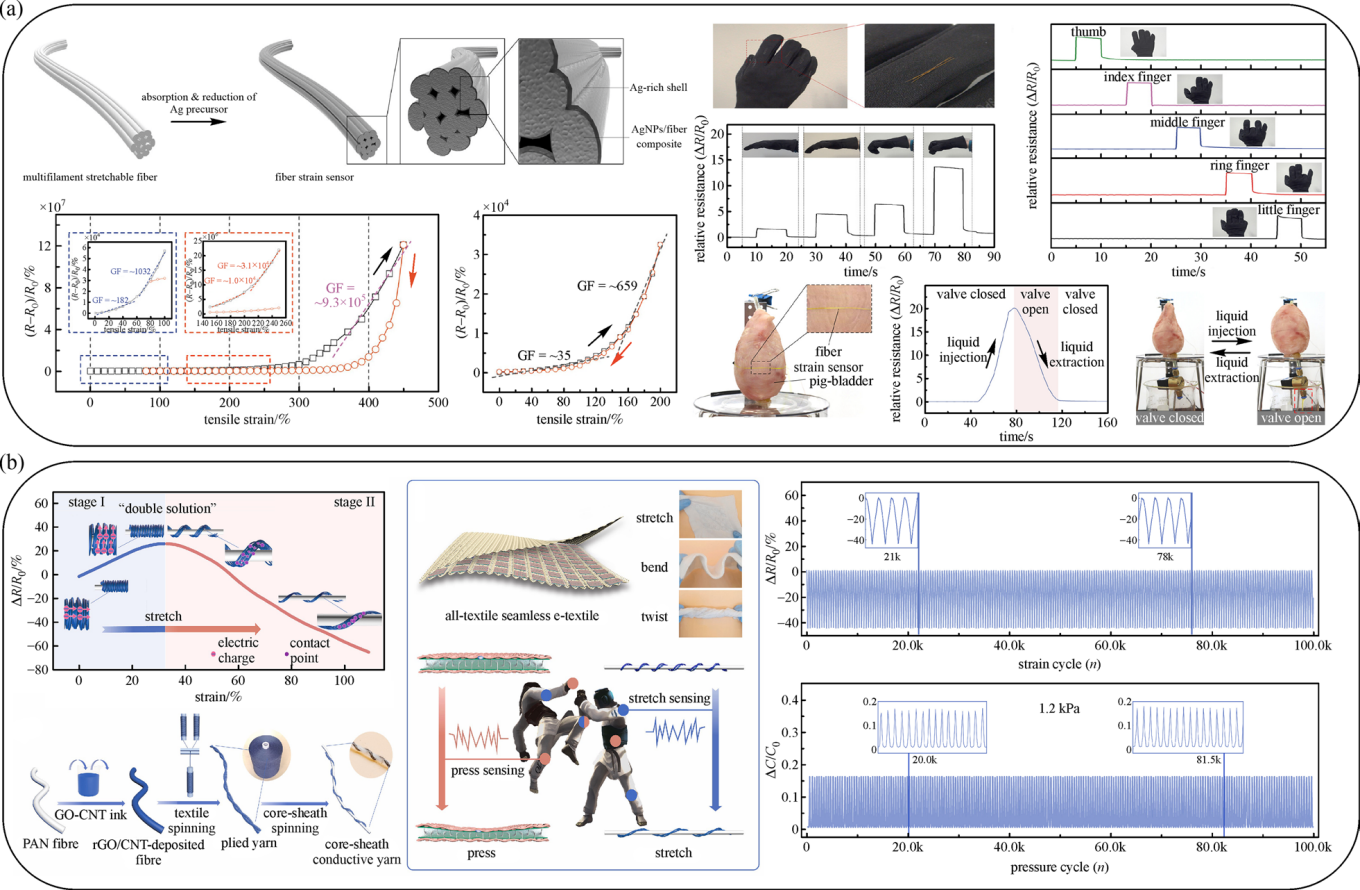
**a** Schematic illustration of the fabrication process, relative resistance changes versus strain in first and subsequent stretching and illustrations of strain sensors integrated into a glove and bladder system. Reproduced with permission [75]. Copyright 2018, American Chemical Society. **b** Conductive yarn fabrication process, schematic graph of e-textile under various bending conditions, and impedance and capacitance response under stretching and pressure respectively during 100 k cycles. Reproduced with permission [76]. Copyright 2021, Elsevier

In most cases, it is extremely difficult to realize a strain sensor with both a wide sensing range and a high gauge factor (GF) simultaneously, which presents a barrier to large deformation detection with high accuracy. One solution has been to utilize silver nanoparticles in the multifilament fiber [75]. The Ag precursor was absorbed by PU fiber and the Ag nanoparticle (AgNP) shell was deposited on the fiber surface to form AgNPs/fiber composite as shown in Fig. 4a. The repetitive absorption and reduction of Ag precursors led to an enhancement of incorporation with polymeric fibers, thereby improving the electrical conductivity. This fiber strain sensor exhibited an excellent GF of ${\text{9.3}} \times 10^{5}$ and 659 in maximum during first and subsequent stretching respectively and a wide sensing range of 450% [75]. Furthermore, the fiber was knitted into a glove and integrated into an artificial bladder system, which demonstrated highly precise detection of human motion and volumetric change.

Recently, the public has shown great enthusiasm about participating in sports activities and competitions. Followed the increasing interest, sportswear embedded with sensors is designed to record data for healthcare monitoring and grading in competitions. However, in such an intense environment, the stability of wearable sensory systems faces tremendous challenges. To overcome this issue, a dual-sensing strategy was proposed [76]. The rGO/CNTs fiber was twisted to form a core-sheath conductive yarn with a helical structure and woven into the fabric as shown in Fig. 4b. By filling Ecoflex between two pieces of fabric, dual tactile sensors were formed, which could be used due to impedance and capacitive variations under stretching and pressing [76]. After more than one hundred thousand cycles, the integrated sensory system still demonstrated high consistency and durability with a deformation range of 90% and heavy strikes of 10 kPa, making it usable for taekwondo training [76]. In another example, the assembly of nanowires on fabric surfaces enabled the combination of good flexibility and durability as well as low resistance [77]. As a result, such an assembly has broad applications in wearable electronics. To illustrate the superiority of mechanical reliability and electrical performance on flexible devices, a fiber piezoresistive sensor was presented. The cotton fiber coated with rGO doped with AgNWs through self-assembly, reduction, and wrapping demonstrated an optimized sensitivity (approximately 4.23 ${\text{kPa}}^{-1}$), the fast response time (0.22 s) and recovery (0.42 s) compared with undoped samples, proving that both mechanical and electrical performance was significantly improved by the uniformly dispersed AgNW.

### Fiber-based logic circuits

**Fig. 5:**
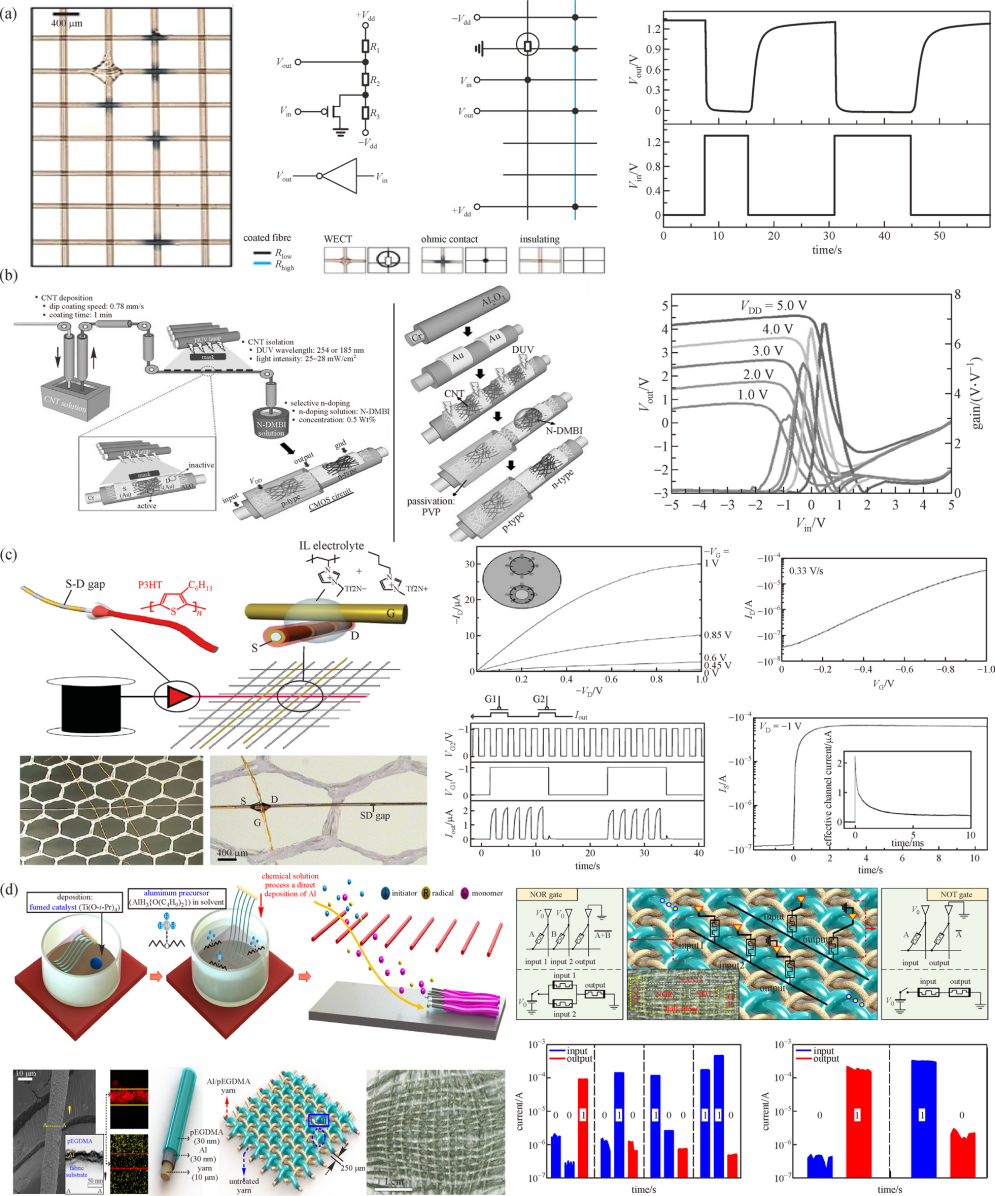
Schematic illustration of **a** classic circuit diagram of an inverter and equivalent circuit on fiber circuit diagram. Reproduced with permission [35]. Copyright 2007, Springer Nature. **b** Full fabrication process, voltage transfer and gain characteristics of fiber-type CMOS circuitry. Reproduced with permission [16]. Copyright 2017, Wiley–VCH. **c** P3HT coating with patterned source-drain gaps and electrical characteristics of the transistor and AND operation. Reproduced with permission [42]. Copyright 2009, Wiley–VCH. **d** Fabrication procedure of memristors on fabrics and operation of NOR and NOT gate. Reproduced with permission [79]. Copyright 2017, American Chemical Society

To achieve fully integrated e-textile systems, it is essential to develop logic and computing components that are recognized as the “brain” of electronic systems for computing and processing. Recently, several approaches have been proposed to realize logic functions on fabrics. Some patterned structures via printing methods have been successfully applied on various organic substrates to fabricate wearable electronics [78]. An alternative method is weaving designated conductive fibers into fabric. For example, inverters, as a fundamental component in logic functions, were demonstrated as shown in Fig. 5a, b. By adding electrolytes, conductive inks, and insulators at the junction of crossly placed PEDOT:PSS coated fibers, an electric inverter was formed. With the help of a fabric circuit diagram, a more complex digital design namely a 4-channel line multiplexer was illustrated, proving that it was possible to design the desired logic operations on fabric substrates [35]. Another example was use of a CMOS inverter on 1D fiber substrates [16, 80]. A fiber transistor was fabricated by CNT deposition, CNT isolation, and subsequent selective doping. Channel isolation and n-type dopant with (4-(1,3-dimethyl-2,3-dihydro-1H-benzimidazole-2-yl) phenyl)-dimethylamine (N-DMBI) improved the ON/OFF ratio and converted the active channel into n-type. The transistor exhibited p-type and n-type electrical mobility of 4.03 and 2.15 ${\text{cm}}^{{2}} {/}\left( {{\text{V}} \cdot {\text{s}}} \right)$ respectively and good dynamic logic operation [16].

Apart from inverters, other logical functions were demonstrated on textiles as shown in Fig. 5c, d [42, 79]. In Fig. 5c, these fibers coated with conductive and organic semiconductor layers were woven into fabric mesh, and the source and drain gap was formed by evaporation using upper fiber as a mask. This transistor exhibited an output characteristic similar to that of p-type MOSFET and the electrical performance at megahertz was also tested. By cascading two adjacent transistors, a two-binary AND gate was presented. The combination of field-effect and electrochemical operation enabled large current at relatively high speed with the compatible textile-production method [42]. One logic-in-memory circuit composed of a series of memristors was achieved on real cotton yarns [79]. Each Al/pEGDMA-coated memristor was cross-linked with each other by inserting one raw yarn to form a memristor crossbar array. The switching between the high-resistance state (HRS) and low-resistance state (LRS) of each memristor enabled basic logic operation like NOT, NOR, and NAND gates, and made it possible to form the large-area integrated circuit on fabrics.

However, there are still some challenges in the field of fabric circuitry that need to be addressed. First, considering the low carrier mobility of organic materials, their electrical performance is severely limited. Moreover, the low diversity of fabric-based electrical components limits high-level integration on textiles. Lastly, stability and robustness are the main concerns in practical applications where failure is possible under large deformation and multiple bending. As a consequence, multi-discipline cooperation is essential to improve the computing ability of e-textiles.

### Fiber-based memory devices

In recent years, the development of information technologies including artificial intelligence (AI), big data, and the Internet of Things (IoT) brings a very large amount of data, which results in big challenges for information processing, computing, and storage. When reducing the size and power consumption of typical field-effect transistors, the conflict between a large amount of data and traditional computer-based von Neumann architecture has become the major challenge for modern electronic systems. Due to the physical limits, there is strong demand for more effective and energy-saving devices with new architecture.

Memory device is a promising candidate for high processing capability and integration density as well as low power consumption. Memristor, a type of resistor with memory effect, was first developed by Leon Chua in 1971 [81] and used to describe the relationship between charge and flex [81]. As a fundamental circuit component, the metal-dielectric-metal two-junction structure shows adjustable resistance modulated by the external voltage, and is thus considered to be suitable for computing devices [82].

**Fig. 6:**
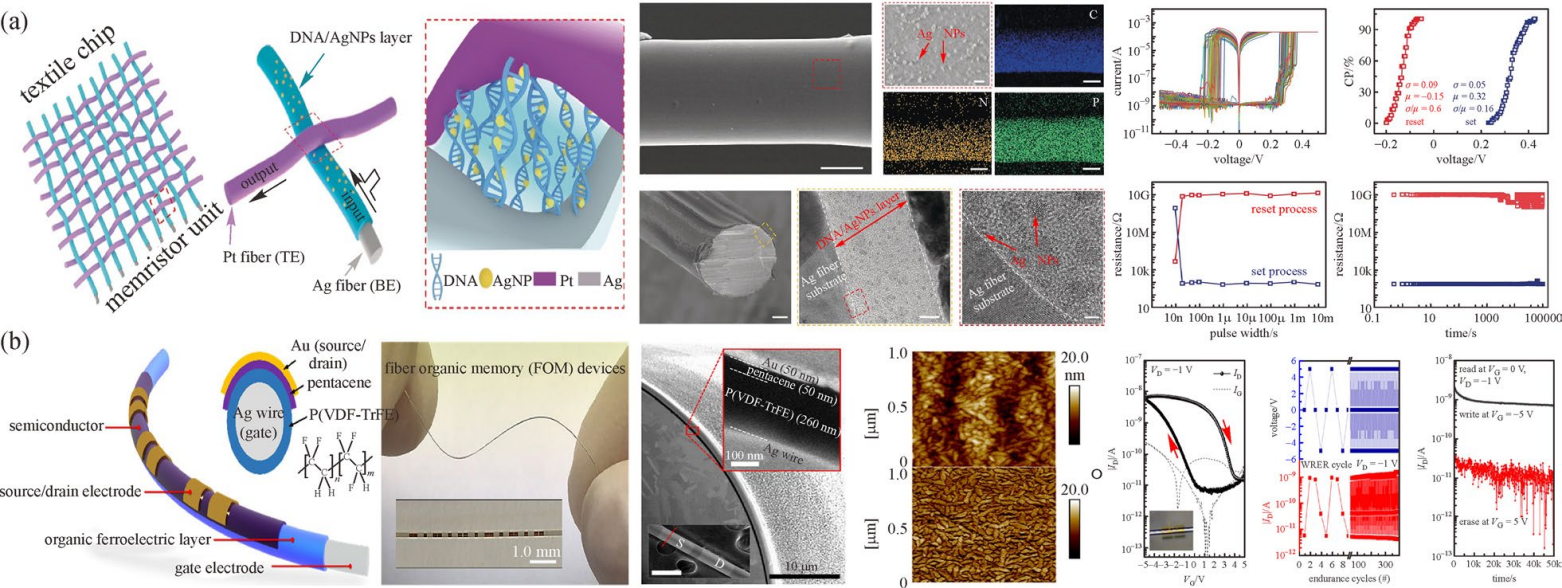
Schematic illustration of **a** a DNA-bridged memristor and memristive characteristics. Reproduced with permission [43]. Copyright 2020, Wiley–VCH. **b** Fabrication process device architecture and *I-V* characteristics of the fiber organic memory. Reproduced with permission [83]. Copyright 2019, American Chemical Society

The fiber-based memristor is also an important element in building e-textile systems. Compared to a planar memristor, its use is even harder to realize due to poor compatibility between the non-planar surface and existing nanofabrication such as lithography and electron beam evaporation. Moreover, the stability between interfaces and the properties of memresistive materials are the main concerns for fiber-based memristors [82]. To address these challenges, a layer of deoxyribonucleic acid (DNA) as active material was successfully coated on fiber electrodes via electrophoretic deposition [43]. In this work, AgNPs and DNA were simultaneously deposited and uniformly distributed along the fiber surface, showing high reliability and superiority of electrophoretic deposition in fiber fabrication. A single memristor consisted of interlaced Ag fiber and Pt fiber with a top-down physical weaving method. A typical memristor switching behavior after 50 voltage sweep cycles, with voltage bias applied, is shown in Fig. 6a [43]. The memristor exhibited fast switching speed within several nanoseconds with a high ON/OFF ratio, low voltage, and energy consumption, which provided a solution to problems of design of novel organic memristors. Besides memristors, some fiber-based memory devices were proposed [17, 84, 85]. One fiber memory was proposed by Kang et al., as presented in Fig. 6b [83]. The ferroelectric copolymer of vinylidene fluoride and trifluoroethylene (P-(VDF-TrFE)) whose polarization states switch reversibly under electrical field was uniformly coated along the entire Ag wire surface through a capillary tube. The low trap energy and fast carrier accumulation in the channel accelerated transfer response and switching speed with high flexibility [83].

### Fiber-based neuromorphic computing

**Fig. 7:**
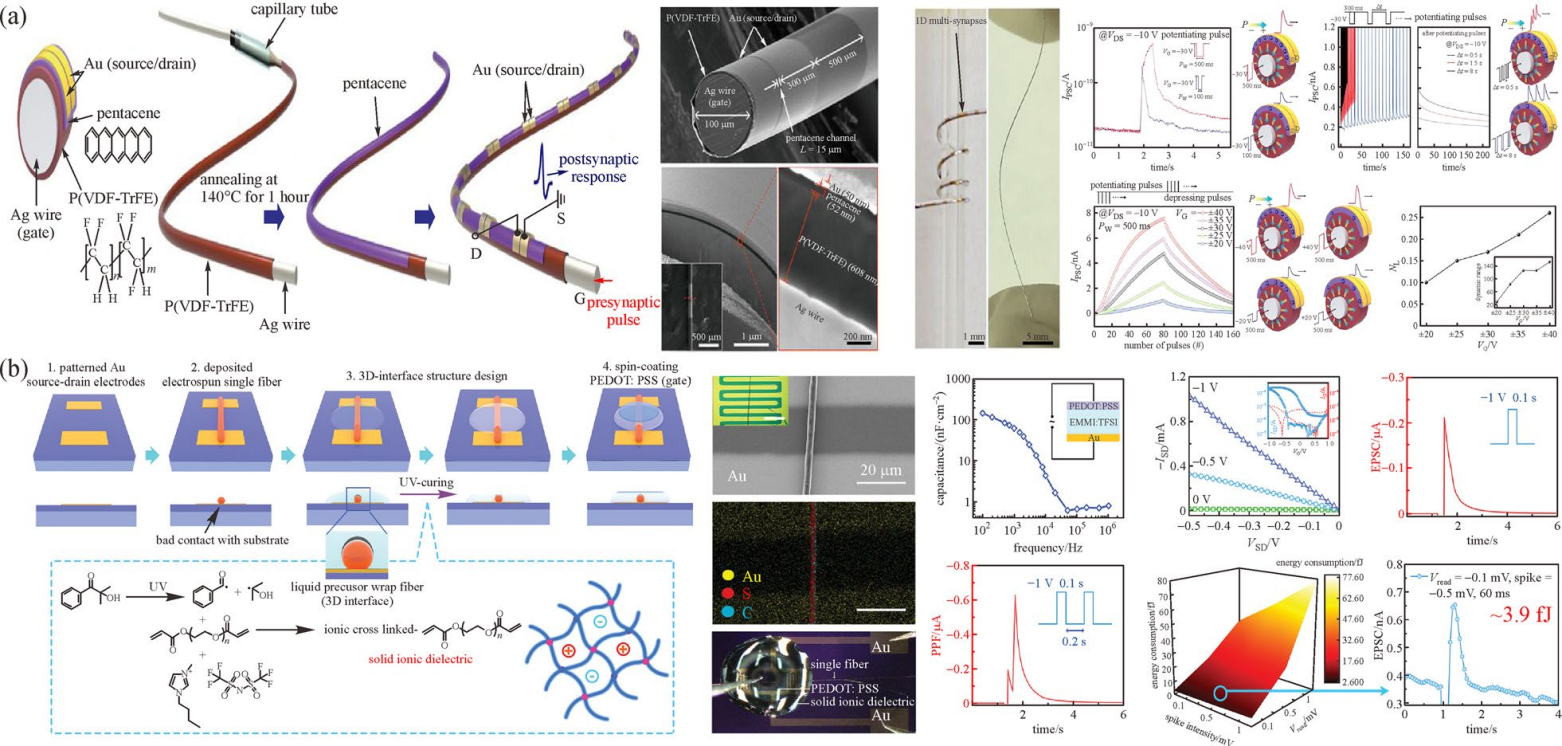
Schematic illustration of **a** fabrication process of the organic artificial multi-synapses, synaptic characteristics and switching mechanism. Reproduced with permission [94]. Copyright 2020, The Authors, published by American Association for the Advancement of Science (AAAS). Reprinted/adapted from Ref. [94]. © The Authors, some rights reserved; exclusive licensee American Association for the Advancement of Science. Distributed under a Creative Commons Attribution NonCommercial License 4.0 (CC BY-NC). **b** Fabrication process device architecture and synaptic characteristics of the single-fiber synaptic transistor. Reproduced with permission [96]. Copyright 2020, Wiley–VCH

Led by the information revolution, neuromorphic computing has attracted enormous attention in recent decades. The brain-inspired technology mimics biological functions using specialized functional devices. Inside the human brain, there are more than $10^{11}$ neurons and 1000 synaptic connections. The neurons can collect and accumulate charges transported through synaptic connection until the total charge exceeds the threshold and then transmit a signal to another neuron while synapses use local memory to determine the pass or suppression of the transmitted signal [86]. Memristors provide high electrical performance and low energy consumption. As a result, the strategy of using memristors to mimic synapse action is widely accepted [87–89]. Apart from memristors, electronic devices which are dedicated to synaptic mimic are known as synapse transistors. To date, synapse transistors have been reported on multiple substrates and operations [90–95]. Organic field-effect transistors, with the advantages of high flexibility and compatibility of large-scale manufacturing with low cost, have become one of the most promising candidates for synaptic devices. For instance, an organic transistor on Ag wire using poly (vinylidene fluoride-trifluoroethylene) (P(VDF-TrFE)) as gating dielectric layer fabricated via dip-coating through a capillary tube was proposed [94]. Au electrodes were thermally deposited on the fiber surface and separated by a distance from each other to form multi-synaptic structures. After the signal transmitted from preneuron propagated along the fiber surface, the received responses were modulated by synaptic weight between multi-postsynaptic, which was generated by pentacene conductance at each synapse. The synaptic characteristic was presented in Fig. 7a. By changing the properties of input pulses (number of cycles and strength), the synaptic weight was controlled, thus modulating the response waveform and amplitude at each synaptic [94]. However, the energy consumption of synaptic transistors has become the main concern due to the amplified power consumption in artificial neuromorphic networks [97, 98]. To reduce the energy consumption, one 3D single-fiber synaptic transistor with a solid-ion gate and ultra-low reading voltage was demonstrated, as shown in Fig. 7b [96]. Electro-spun fiber as the active channel was placed on the top of patterned Au electrodes and wrapped by a liquid electrolyte through solidification to ensure electrical contact at the interface. Ionic transportation induced by a small presynaptic spike generated a conducting channel on the fiber surface and enabled the electrical response similar to synapse operation [96]. This artificial synapse exhibited advantages of low-voltage operation and high sensitivity, and thus showed potential in integrated neuromorphic computation systems.

## Conclusions and outlook

In this review, we have discussed the features, potentials, and challenges of fiber-based transistors and their applications in sensing, logic, memory, and neuromorphic computation using e-textile systems. Textile-based electronics has been well-developed and has achieved technological progress with high electrical performance and stretchability, lightweight potential, and flexibility. In addition, several other fiber-based devices like actuators and light-emitting diodes are under intensive study. There is a rapidly increasing effort of research and investigation into wearable electronics based on 1D fiber and 2D fabrics all over the world. Recently, the focus on achieving high-performance individual electronic units has been transferred to realization of system-level applications on fabrics. As a consequence, partially integrated e-textile systems have been developed on multiple substances for wearable electronics [99–101]. However, the realization of fully integrated e-textile systems is still difficult due to the limitation of the current technologies in building more functional blocks, such as ADC, data processors, and wireless communications. The types of electronic components adapted in textiles are still insufficient to accomplish large-scale integrated systems. Therefore, multidisciplinary cooperation integrating material science, physics, microelectronics, and big data are essential to enhance functionalities and integrity for e-textiles. The next-generation e-textile systems may have the potential to demonstrate high compatibility for embedding a variety of modern electronic devices to enhance the capability of signal processing, computing, and storage. Then, fully integrated e-textile systems with high mechanical and environmental stability and minimum power consumption will become promising candidates for healthcare, sports, and entertainment applications.
